# Multimodality imaging fusion to guide stereotactic radioablation for refractory complex ventricular tachycardia

**DOI:** 10.1016/j.hrcr.2022.09.008

**Published:** 2022-09-16

**Authors:** Alba Santos-Ortega, Nuria Rivas-Gándara, Gabriel Pascual-González, Alejandro Seoane, Raquel Granado, Victoria Reyes

**Affiliations:** ∗ Arrhythmia Unit, Cardiology Department, Vall d’Hebron University Hospital, Barcelona, Spain; † Faculty of Medicine and Surgery, Universitat Autonoma Barcelona (UAB), Barcelona, Spain; ‡ Medical Physics Department, Vall d’Hebron University Hospital, Barcelona, Spain; § Radiation Oncology Department, Vall d’Hebron University Hospital, Barcelona, Spain

**Keywords:** Stereotactic radioablation, Ventricular tachycardia, Intramural ventricular tachycardia, Hypertrophic cardiomyopathy, Multimodality imaging

## Introduction

Ventricular tachycardia (VT) is associated with a high risk of sudden cardiac death (SCD). The widespread implantation of defibrillators as an SCD prevention strategy has increased the number of survivors of VT or ventricular fibrillation. These arrhythmias represent a difficult-to-treat problem of great magnitude, with catheter ablation currently being the most accepted therapy.^1^ However, despite technical advances in electroanatomical mapping (EAM) and new ablation catheters, the arrhythmic recurrence rate is approximately 50%, exposing patients to multiple invasive procedures and potentially deleterious defibrillator shocks.^2^

Stereotactic radiotherapy (SRT) is a widely used treatment in oncology whose mechanism of cell death involves apoptosis and microvascular damage. Technical advances have allowed the administration of radiation with great precision and are highly effective, with few side effects. In recent years, SRT has emerged as a promising alternative for the treatment of refractory ventricular arrhythmias. Studies published to date have shown the high efficacy of this therapy in reducing arrhythmic events, up to 94%, with a low rate of complications.^3^, ^4^, ^5^ SRT should be guided by imaging techniques, and treatments applied to the heart have a delayed effect.

## Case report

Here we report the case of a 71-year-old patient with a medical history of hypertension, dyslipidemia, overweight, and endothoracic multinodular goiter with normal thyroid function. After SCD was diagnosed in 1 of his siblings, a family study was conducted, from which he was diagnosed with hypertrophic obstructive cardiomyopathy, observing a mutation in *MYBPC3* p.Glu542Gln. Cardiac magnetic resonance imaging (MRI) revealed severe asymmetric septal hypertrophy (maximum septal thickness, 31 mm), preserved systolic function, and an extensive and diffuse intramyocardial area of fibrosis in the anterior wall, apical segments, and junction of the left and right ventricles. In January 2019, a single-chamber defibrillator was implanted for prevention.

**Figure 1:**
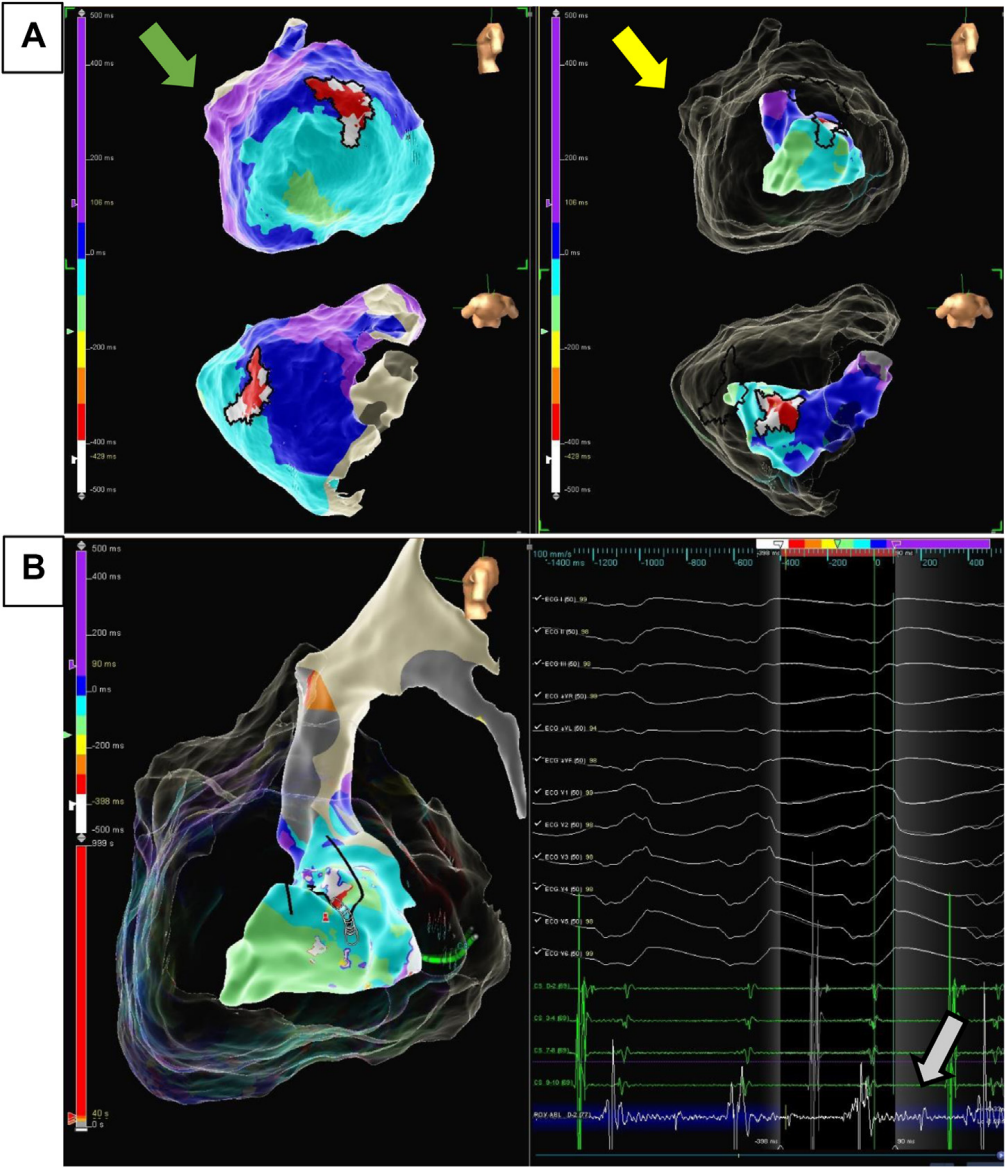
Electroanatomical activation mapping during ventricular tachycardia with Abbott EnSite Precision (Abbott, Minneapolis, MN). **A:** The left image (*green arrow*) shows epicardial mapping. The right image (*yellow arrow*) shows endocardial mapping. A significant part of the tachycardia cycle is not represented on any of the electroanatomical maps, suggesting an intramyocardial origin of the tachycardia. **B:** The left part of the image shows endocardial mapping. On the right, electrocardiogram showing ventricular tachycardia with right bundle brunch block morphology and a superior axis suggestive of an anterolateral left ventricle origin. Low-voltage mesodiastolic electrograms (*gray arrow*), suggestive of far-field signals, are found on catheter recordings during endocardial mapping, supporting an intramyocardial origin.

One year later, he was admitted to the cardiac intensive care unit with an arrhythmic storm that occurred with multiple defibrillator therapies. Coronary angiography ruled out coronary obstruction. Medical treatment with intravenous procainamide and esmolol and subsequent amiodarone was ineffective. The patient was subsequently referred for cardiac ablation. Considering the transmural substrate predicted by MRI, both subxiphoid epicardial and double endocardial retroaortic and transseptal access were achieved. Using a multipolar Advisor HD Grid™ catheter and EnSite Precision (Abbott, St. Paul, MN) EAM system, a voltage map was created that showed a very extensive low-voltage area in the left ventricle, predominantly in the anterior, apical, and lateral epicardium, suggestive of epicardial fat. A VT with a cycle length of 530 ms and the same morphology as the clinical VT was induced (Figure 1A). It was clinically well tolerated, which allowed mapping during tachycardia. The activation map showed a macroreentrant circuit in the anterolateral area of the left ventricle with endocardial and epicardial components. However, only 70% of the tachycardia cycle was obtained despite mapping of the epicardium and endocardium, suggesting that the critical isthmus was located intramyocardially (Figure 1B). Extensive radiofrequency ablation during VT was applied from the endocardial and epicardial sides but was ineffective owing to difficult access to this intramyocardial substrate, which was aggravated in this patient with severe myocardial hypertrophy.

**Figure 2:**
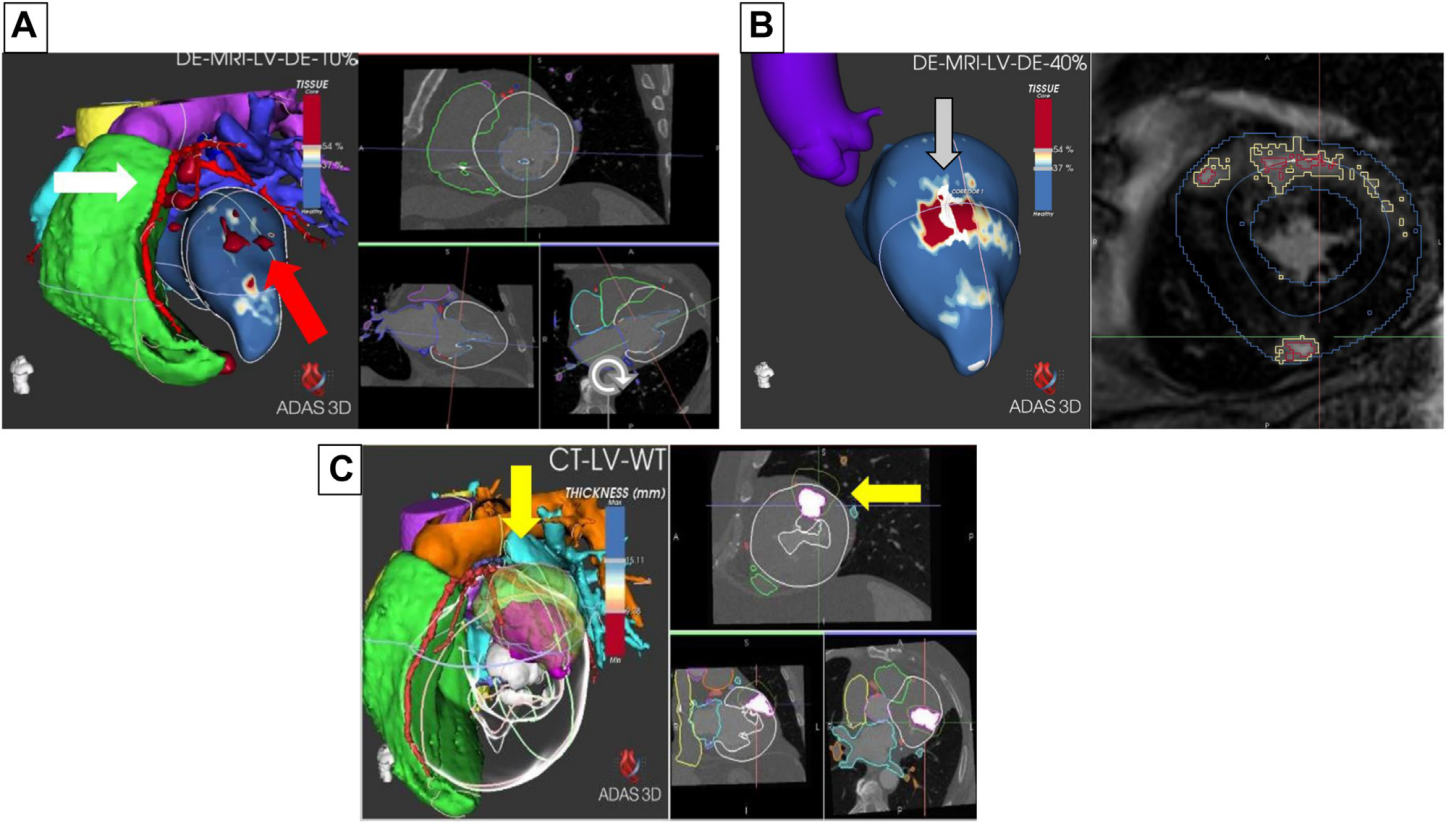
ADAS 3D (Barcelona, Spain) reconstruction. **A:** Cardiac computed tomography (CT) and magnetic resonance imaging (MRI) fusion. The left ventricle is represented in layers corresponding to myocardial depth and this image belongs to the endocardium (10%). Cardiac structures are represented as follows: right ventricle (*green*), pulmonary artery (*purple*), aorta (*yellow*), left atrium (*blue*), and coronary arteries (*white arrow*). Red arrow points out intramyocardial substrate observed in MRI. **B:** MRI reconstruction with ADAS shows intramyocardial layer (40%). Grey arrow points out an intramyocardial corridor, corresponding to ventricular tachycardia critical isthmus. **C:** Cardiac CT / electroanatomical map and radiotherapy planning CT fusion. Area of interest delimited in electroanatomical map is represented (*yellow arrow*).

After evaluating the difficulty of treating VT with conventional techniques owing to its intramyocardial substrate associated with severe ventricular hypertrophy, an alternative approach consisting of noninvasive radioablation (NIRA) was considered. Identification of the target area in this case was challenging because of its intramyocardial nature. Therefore, we used ADAS 3D (Barcelona, Spain) image postprocessing software to plan the procedure. This tool allows the fusion of 3-dimensional (3D) images (MRI and cardiac computed tomography [CT]) with the EAM for correct and precise identification of the area of critical isthmus of the tachycardia. More importantly, it made it possible to avoid other nearby structures (eg, coronary arteries, mitral valve), whose injury could have potential complications (Figure 2A).

To plan radiotherapy treatment, 4-dimensional CT was performed to control for and adjust the respiratory and cardiac movements, and the defibrillator was kept pacing at a heart rate of 100 beats per minute. Using the ADAS 3D tool, it was possible to combine previous MRI and cardiac CT images with the EAM as well as with the radiotherapy planning CT (Figure 2B).

**Figure 3:**
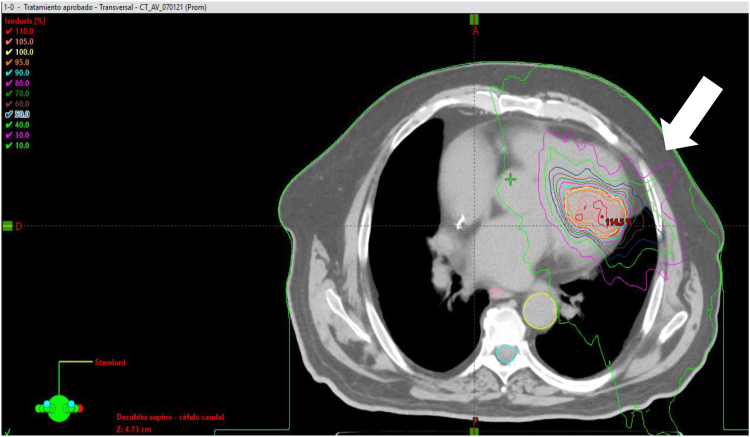
Noninvasive cardiac radioablation treatment using a TrueBeam (Varian Medical Systems) radiotherapy plan showing the target volume (*white arrow*) and isodose lines.

To define the area of interest for NIRA, a clinical target volume was delimited on the fusion image obtained with ADAS 3D software. A margin of 0.3 mm was added to obtain the internal target volume, which considers respiratory movements. Organs at risk included the lungs, trachea, esophagus, stomach, medulla, rib wall, liver, aorta, pericardium, anterior descending coronary artery, left ventricle, and papillary muscle. A 25 Gy session was applied in the TrueBeam® linear accelerator (Varian Medical Systems, Palo Alto, CA), with the RapidArc technique and treatment verification by cone-beam CT (Figure 3), as well as respiratory and heart rate control.

During the acute phase, the patient presented with mild asthenia. The electrocardiogram taken after NIRA showed no significant changes compared to the previous ones. Blood tests performed 8 hours later showed only a minimal increase in cardiac enzymes, which normalized at 24 hours.

Echocardiography performed in the first few hours revealed very slight pericardial effusion with a maximum of 5 mm in the posterior space without hemodynamic compromise of the cardiac chambers.

One year later, the patient remained asymptomatic and presented no new arrhythmic events or relevant side effects related to NIRA.

## Discussion

This is the first reported case in which ADAS 3D image postprocessing software was used to plan an NIRA procedure by fusing cardiac images with radiotherapy CT images. This supports the usefulness of NIRA for refractory ventricular arrhythmia treatment and its promising application in complex arrhythmic substrates assisted by careful multimodal imaging planning.

Advances in the recognition and diagnosis of ventricular arrhythmias have resulted in more complex arrhythmia substrates. Despite the latest advances in catheter ablation, we often encounter great difficulties in treatment in relation to anatomical access and target area identification. Characterization of the 3D VT circuit, as proposed by Tung and colleagues,^6^ showed the presence of intramyocardial reentry in a significant percentage of patients (up to 18%). With conventional EAM systems, accurate identification of the slow conduction channels that cause intramyocardial reentry tachycardias is often challenging. For this reason, the fusion of cardiac CT and MRI images using this image postprocessing software enables definition of the substrate in 3 dimensions. In patients with these types of tachycardias, a better understanding of the tachycardia mechanism and more targeted treatment is favored.

In addition, intramyocardial reentry tachycardias can be inaccessible to conventional ablation catheter treatment, especially in patients with severe myocardial hypertrophy or with a septal origin. Radiotherapy seems to be a promising alternative for facilitating treatment in deeper areas. Studies to date have shown high efficacy of radiotherapy treatment without acute toxicity, with pericarditis and delayed pericardial effusion (28%) and pneumonitis (11%) being the most frequent complications, with good response to medical treatment.^4^ However, follow-up studies are lacking, which is why the long-term effects of this emerging therapy remain unknown. Notwithstanding, the fusion of imaging techniques, EAM, and radiotherapy CT allows the accurate application of radiotherapy, further minimizing possible deleterious effects on nearby cardiac structures (valve apparatus, coronary arteries, etc).

Finally, ADAS 3D postprocessing tool use was previously reported in a case of VT with an inaccessible substrate for endocardial catheter ablation owing to an intraventricular thrombus. In this patient, the analysis of channels in cardiac CT using this software allowed for precise NIRA treatment planning.^7^ Nevertheless, for the first time, in our case, we used the fusion of cardiac images (CT and MRI) and EAM with ADAS 3D, which was also merged with radiotherapy CT. The advantages of highly accurate anatomical definition via the fusion of several imaging techniques increased treatment precision in our case.

## Conclusion

Here we presented a case in which NIRA allowed successful treatment of a patient with a complex arrhythmic substrate that was inaccessible using conventional techniques. This case highlights the usefulness of cardiac image fusion using ADAS 3D postprocessing software merged with radiotherapy CT. It facilitates adequate procedure planning, allowing radiotherapy to be administered with greater accuracy and the least possible short- and long-term side effects.

The need for the accurate identification of target areas for radiotherapy application makes us expect great growth in this technique for other complex arrhythmic substrate treatment applications in which imaging fusion via specific postprocessing tools could play a relevant role.

**Key Teaching Points**

• Multimodal imaging technique fusion using ADAS 3D (Barcelona, Spain) software enables precise noninvasive radioablation planning.
• Noninvasive radioablation is an emerging technique that is becoming a promising alternative for refractory ventricular arrhythmia treatment.
• Ventricular arrhythmias due to intramyocardial reentry require special therapeutic management in which noninvasive radioablation may play a relevant role.

## References

[1] Priori S.G., Blomström-Lundqvist C., Mazzanti A. (2015). 2015 ESC Guidelines for the management of patients with ventricular arrhythmias and the prevention of sudden cardiac death: The Task Force for the Management of Patients with Ventricular Arrhythmias and the Prevention of Sudden Cardiac Death of the European Society of Cardiology (ESC). Endorsed by: Association for European Paediatric and Congenital Cardiology (AEPC). Eur Heart J.

[2] Cronin E.M., Bogun F.M., Maury P. (2019). 2019 HRS/EHRA/APHRS/LAHRS expert consensus statement on catheter ablation of ventricular arrhythmias. Europace.

[3] Cuculich P.S., Schill M.R., Kashani R. (2017). Noninvasive cardiac radiation for ablation of ventricular tachycardia. N Engl J Med.

[4] Zei P.C., Soltys S. (2017). Ablative radiotherapy as a noninvasive alternative to catheter ablation for cardiac arrhythmias. Curr Cardiol Rep.

[5] Robinson C.G., Samson P.P., Moore K.M.S. (2019). Phase I/II trial of electrophysiology-guided noninvasive cardiac radioablation for ventricular tachycardia. Circulation.

[6] Tung R., Raiman M., Liao H. (2020). Simultaneous endocardial and epicardial delineation of 3D reentrant ventricular tachycardia. J Am Coll Cardiol.

[7] Zucchelli G., Parollo M., Di Cori A., Matteucci F., Berruezo A., Bongiorni M.G. (2021). Stereotactic ventricular tachycardia radioablation aided by CT-channels analysis in a patient with inaccessible transmural substrate. Europace.

